# Lean methodology: contributions to improving work processes in health and nursing

**DOI:** 10.1590/0034-7167-2023-0322

**Published:** 2024-05-13

**Authors:** Fernanda Novaes Moreno Brancalion, Lara Gonçalves de Souza, Simone Berger, Antônio Fernandes Costa Lima

**Affiliations:** I Universidade de São Paulo. São Paulo, São Paulo, Brazil; II Universidade de São Paulo, Escola Politécnica. São Paulo, São Paulo, Brazil

**Keywords:** Total Quality Management, Efficiency, Organizational, Cost Efficiency Analysis, Process Optimization, Cost Savings, Gestión de la Calidad Total, Eficiencia Organizacional, Análisis Costo-Eficiencia, Optimización de Procesos, Ahorro de Costo, Gestão da Qualidade Total, Eficiência Organizacional, Análise de Custo-Eficiência, Otimização de Processos, Redução de Custos

## Abstract

**Objective:**

to investigate the contributions of applying the Lean methodology to improve work processes in health and nursing and its impact on associated financial aspects.

**Method:**

an integrative review, carried out in six databases, whose sample of ten (100.0%) studies was analyzed and summarized descriptively.

**Results:**

the outcomes obtained were stratified into: benefits/barriers to Lean Healthcare implementation; economic aspects involving Lean Healthcare implementation; and process improvements through Lean Healthcare implementation. The majority of studies (60.0%) were carried out in university hospitals, contexts that need to continually improve the quality of services provided, generally with scarce and limited resources, which support the viability of maintaining the teaching, research and extension tripod.

**Conclusion:**

three (30.0%) studies highlighted the financial aspects associated with Lean methodology application. The others only mentioned the possibility of financial gains through improving processes and reducing waste.

## RESUMO

**Objetivo:**

investigar as contribuições da aplicação da metodologia *Lean* para melhoria dos processos de trabalho em saúde e enfermagem e sua repercussão nos aspectos financeiros associados.

**Método:**

revisão integrativa, realizada em seis bases de dados, cuja amostra de dez (100,0%) estudos foi analisada e sintetizada descritivamente.

**Resultados:**

os desfechos obtidos foram estratificados em: benefícios/barreiras para implantação do *Lean Healthcare*; aspectos econômicos envolvendo a implantação do *Lean Healthcare*; e melhorias em processos por meio da implantação do *Lean Healthcare*. A maioria dos estudos (60,0%) foi realizada em hospitais universitários, contextos que precisam melhorar, continuamente, a qualidade dos serviços prestados, geralmente com recursos escassos e limitados, os quais sustentam a viabilidade da manutenção do tripé ensino, pesquisa e extensão.

**Conclusão:**

três (30,0%) estudos evidenciaram os aspectos financeiros associados à aplicação da metodologia *Lean*. Os demais apenas mencionaram a possibilidade de ganhos financeiros por meio da melhoria de processos e redução de desperdícios.

## RESUMEN

**Objetivo:**

investigar las contribuciones de la aplicación de la metodología Lean a la mejora de los procesos de trabajo en salud y enfermería y su impacto en los aspectos financieros asociados.

**Método:**

revisión integradora, realizada en seis bases de datos, cuya muestra de diez (100,0%) estudios fue analizada y resumida de forma descriptiva.

**Resultados:**

los resultados obtenidos se estratificaron en: beneficios/barreras para la implementación de Lean Healthcare; aspectos económicos que implican la implementación de Lean Healthcare; y mejoras de procesos mediante la implementación de Lean Healthcare. La mayoría de los estudios (60,0%) se realizaron en hospitales universitarios, contextos que necesitan mejorar continuamente la calidad de los servicios prestados, generalmente con recursos escasos y limitados, que sustentan la viabilidad de mantener el trípode de docencia, investigación y extensión.

**Conclusión:**

tres (30,0%) estudios destacaron los aspectos financieros asociados a la aplicación de la metodología Lean. Los demás solo mencionaron la posibilidad de obtener ganancias financieras mejorando los procesos y reduciendo el desperdicio.

## INTRODUCTION

Lean methodology consists of a fact-based and data-driven improvement philosophy that values preventing defects over detecting them, aiming at customer satisfaction through delivering the best quality at the lowest possible cost^(1)^. Of industrial origin, its principle has been applied in the health area, due to the similarity of the production processes of institutions that, despite the distinctions, they seek to plan and execute actions, in a determined time, with specific resources, at the lowest cost, to create value for a customer^(2)^.

Lean aimed to identify waste, eliminating what does not add value (NAV) to customers, in order to increase process efficiency and product quality. In lean processes, added value is obtained through flows that include the essential activities that the customer goes through, and during the execution of these activities, value is added. Thus, added value results from cycles of continuous improvements, which are sources of innovation^(3)^.

In the health area, this methodology is called Lean Healthcare, whose results include identifying and eliminating waste in production processes, with the main focus on adding quality and delivering to customers only what they consider to be value (services that respect and meet their preferences and needs), through an efficient and waste-free process^(2-4)^.

Lean Healthcare adds maturity to management, as it goes beyond departmental vision and creates a process-oriented culture that can encourage people’s engagement to deliver benefits to the business or the institutional mission and vision. Therefore, management needs to be clear about the final results it wants to achieve, assigning those responsible for the sustainability of processes and measuring performance based on these^(2)^.

Lean Healthcare application, as it is based on formal instructions and standardization of the work environment, professional training and respect for people, is favorable for increasing quality of care and patient safety. It assumes that “front line” or production chain professionals are able to assess and contribute to what patients need to have their needs met, considering good clinical practices. In this way, the strategic level approaches the production chain and effectively manages the practice that will progressively impact strategic indicators^(2)^.

The main repercussions arising from the application of this methodology are associated with increased team productivity and efficiency, reduced patient waiting time for care, standardization of care processes and, consequently, reduced costs^(2)^.

Considering that the result of the production process is dependent on people’s behavior, in addition to the focus on standardizing activities, which provide greater productivity, it is necessary to have in-depth knowledge of current work procedures, with a view to continuous improvements in the process. Another important element is the institutional administrative commitment to investing in and promoting a culture of continuous improvement, covering a set of principles and personal values that transform people’s way of life, guiding them towards improvement-seeking behavior with investment in corporate education, competency-based management and indicators^(3)^.

It is noteworthy that Lean thinking underlies a management model that is a reference in terms of operational excellence and the achievement of quality care, through the improvement of processes, with the central aspects that patients value, such as better, safe, fast, qualified and resolute service^(5)^.

The achievements of implementing continuous improvement strategies are recognized around the world. Using such strategies, processes are improved and efficiency increases and, therefore, waste is reduced and savings can be passed on to consumers, resulting in improved quality. When customers receive quality products, companies are able to increase their revenue and, consequently, the economy grows^(6)^.

It is noteworthy that, in Brazil, resources allocated to the health sector are increasingly scarce and limited, and the commitment of leaders, regardless of the legal nature of the institutions (public, private or philanthropic), with the organizational management model, cost management and its repercussions on the viability of different work processes is essential to ensure the provision of safe, efficient, effective and financially sustainable health services^(7)^.

Furthermore, Lean requires a structural change, from top management to the production chain, not being a point of arrival in itself, but rather a path of continuous search for improvements and operational excellence^(7)^. In health service provision, this methodology is designed to create a culture that applies science to design, execute and continually improve the work provided, focusing on measurable value to patients.

## OBJECTIVE

To investigate the contributions of applying the Lean methodology to improve work processes in health and nursing and its impact on associated financial aspects.

## METHODS

### Ethical aspects

Due to the type of article (review), there is no need for approval from a Research Ethics Committee.

### Study design, place, and data collection period

This integrative review, a method that provides the synthesis of knowledge and the incorporation of the applicability of results from significant studies in practice^(8)^, was conducted based on the guiding question: what are the contributions of applying Lean methodology to improve work processes in health and nursing and its impact on associated costs?

The PICO strategy was used, an acronym for Population, Intervention, Comparison and Outcomes^(9)^, using the letters and their equivalent terms: “P” - work processes in health and nursing; “I” - Lean methodology application; “C” - no intervention was established for comparison; and “O” - improvement of processes and associated financial aspects.

To ensure the rigor of this review, the following steps were taken^(9)^: objective establishment; inclusion and exclusion criteria establishment (sample selection); definition of information to be extracted from selected articles; analysis of results; presentation and discussion of results. To select the articles, databases and portals were used that allowed expanding the scope of research^(10)^.

### Sample; inclusion and exclusion criteria

Articles published in Portuguese, English and Spanish, with free texts available, in full, in the selected databases, in the period between 2018 and 2022, which highlighted the contributions of applying Lean methodology in health/nursing work processes and their repercussions on financial aspects, were included. Articles available only in abstract format, published only in conference annals and with paid access were excluded.

### Study protocol

Based on the guiding question, a search was carried out in the US National Library of Medicine (PubMed), Latin American and Caribbean Center for Health Sciences Information (LILACS), Scientific Electronic Library Online (SciELO), Cumulative Index to Nursing & Allied Health Literature (CINAHL), Scopus, Virtual Health Library (VHL) and Engineering Village databases.

Controlled descriptors were delimited (MeSH and MeSH terms, CINAHL Headings, Engineering Village Headings and DeCS) as well as specific keywords for some databases and their derivatives. They were combined with the appropriate Boolean operators (OR and AND). The Boolean operator OR was used between descriptors from the same PICO group, and AND, to search between the PICO groups, being:

- PubMed (MeSH): Nursing OR Nursing Team OR personnel management hospital OR Job description OR professional role AND Total quality management OR value based purchasing OR workflow AND Hospital Costs OR cost and cost analysis OR Health Care Economics and Organizations OR practice valuation and purchase OR cost savings;
- CINAHL (CINAHL term): Team Nursing OR Nurse Administrator role OR personnel management hospital OR Job description OR professional role AND Total quality improvement in nursing OR quality management OR value based purchasing OR value based insurance OR workflow OR workflow analysis OR workflow analysis in nursing AND Health care costs OR costs and cost analysis OR cost control OR nursing costs OR economic aspects of illness OR cost savings;
- VHL (DeCS): *enfermagem* OR nursing OR *enfermería* OR “*equipe de enfermagem*” OR” nursing team” OR “*grupo de enfermería*” OR “*administração de recursos humanos hospital*” OR “personnel administration hospital” OR “*administración de personal en hospitales*” OR “*descrição de cargo*” OR “job description” OR “*perfil laboral*” OR “*papel profissional*” OR “professional role” OR *rol profesional* AND “*gestão qualidade total*” OR “total quality management” OR “*gestión de la calidad total*” OR “*aquisição baseada em valor*” OR “value-based purchasing” OR “*compra basada em calidad*” OR “*fluxo de trabalho*” OR workflow OR “*flujo de trabajo*” AND “*custos hospitalares*” OR “hospital costs” OR “*costos de hospital*” OR “*análise de custos economia e organizações dos cuidados em saúde*” OR “costs and cost analysis” OR “*costos y análisis de costo*” OR “*determinação do valor econômico de organizações de saúde*” OR “practice valuation and purchase” OR “*valorización y adquisición práctica*” OR “*redução de custos*, cost savings*, ahorro de costo”;*
- Scopus (keyword): Nursing OR Nursing Team OR personnel management hospital OR Job description OR professional role AND Total quality management OR value based purchasing OR workflow AND Costs OR cost and cost analysis OR health care economics and organizations OR practice valuation and purchase;
- SciELO (DeCS): *enfermagem* OR nursing OR *enfermería* OR “*equipe de enfermagem*” OR “nursing team” OR “*grupo de enfermería*” OR “*administração de recursos humanos hospital*” OR “personnel administration hospital” OR “*administración de personal en hospitales*” OR “*descrição de cargo*” OR “job description” OR “*perfil laboral*” OR “*papel profissional*” OR “professional role” OR *rol profesional* AND “*gestão qualidade total*” OR “total quality management” OR “*gestión de la calidad total*” OR “*aquisição baseada em valor*” OR “value-based purchasing” OR “*compra basada em calidad*” OR “*fluxo de trabalho*” OR workflow OR “*flujo de trabajo*” AND “*custos hospitalares*” OR “hospital costs” OR “*costos de hospital*” OR “*análise de custos economia e organizações dos cuidados em saúde*” OR “costs and cost analysis” OR “*costos y análisis de costo*” OR “*determinação do valor econômico de organizações de saúde*” OR “practice valuation and purchase” OR *“valorización y adquisición práctica”* OR “*redução de custos*” OR “cost savings” OR *“ahorro de costo”*;
- Engineering Village (keyword): Nursing OR Human Resource Management OR job Analysis OR job description OR professional role AND Quality control OR lean production OR manufacture OR process monitoring OR lean six sigma OR work simplification OR aggregate value OR aggregates OR concrete aggregates AND Cost OR cost benefit analysis OR optimization OR hospitals OR cost effectiveness OR economics.

### Analysis of results

Data originating from the sample of selected studies were analyzed and synthesized descriptively.

## RESULTS

**Figure 1:**
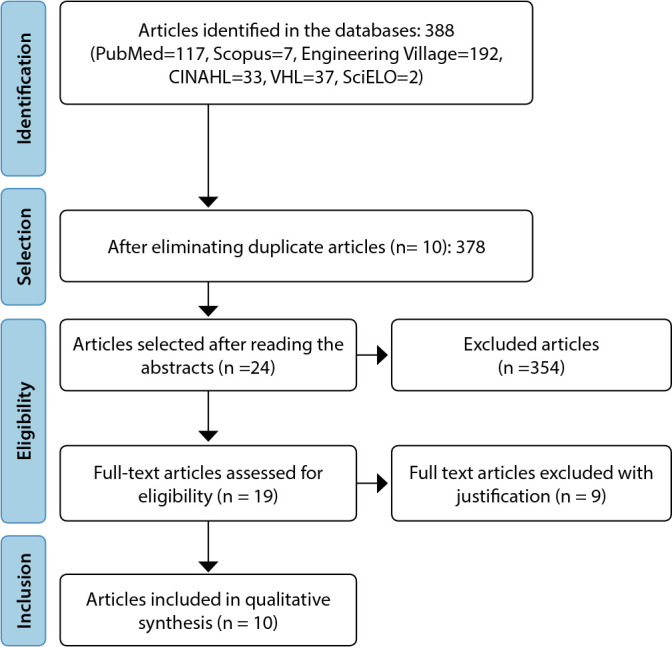
Flowchart of the primary studies selection process adapted from the Preferred Reporting Items for Systematic Review and Meta-Analyses (PRISMA), 2023

According to Figure 1, from January 2018 to October 2022, 378 primary studies were identified in the previously described databases. During the selection, 10 duplicate (identical) articles were eliminated and 354 articles that did not meet the inclusion criteria were excluded. The full texts of 19 eligible articles were assessed, obtaining a sample of 10 articles^(7,11-19)^.

The 10 articles analyzed^(7,11-19)^ were published in the English language, two with complete translation into Portuguese, according to Chart 1. Studies in journals from the United States of America (50.0%) and Brazil (20.0%) predominated. The years of greatest publication corresponded to 2020 (30.0%), 2021 (20.0%) and 2022 (30.0%).

**Chart 1:** Characterization of the 10 articles included in the integrative review according to citation, title, year, country and journal, 2023

Citation	Title	Year	Country	Journal
^(11)^	The sustainability of Lean in pediatric healthcare: a realist review	2018	United Kingdom	Systematic reviews
^(12)^	Process optimization in total knee arthoplasty procedures: Impact of size-specific instrument sets on costs and revenue	2019	Germany	Orthopade
^(13)^	Endoscopic Radial Artery Harvesting During Anesthesia Line Placement Reduces the Time and Cost of Multivessel Coronary Artery Bypass Grafting	2020	USA	Innovations (Phila)
^(14)^	A comparative study using time-driven activity-based costing in single-fraction breast high-dose rate brachytherapy: An integrated brachytherapy suite vs. decentralized workflow	2020	USA	Brachytherapy
^(15)^	Lean Healthcare in the institutional, professional, and patient perspective: an integrative review	2020	Brazil	*Revista Gaúcha de Enfermagem*
^(16)^	Value Improvement by Assessing IR Care via Time-Driven Activity-Based Costing	2021	USA	Journal of Vascular and Interventional Radiology
^(17)^	Lean Healthcare Tools for Processes Evaluation: An Integrative Review	2021	Switzerland	International journal of environmental research and public health
^(18)^	Application of PDCA Process Management in Day Operation Ward and the Influence of Nursing Quality and Safety	2022	USA	Computational and Mathematical Methods in Medicine
^(19)^	The Supervision and Management Mode of Disinfection Supply Center Improves the Standardization of Sterile Goods Management in Clinical Departments	2022	USA	Computational and Mathematical Methods in Medicine
^(7)^	Process-based management aimed at improving health care and financial results	2022	Brazil	*Revista da Escola de Enfermagem da USP*

It was found that, although all articles in the sample relate Lean methodology application to the impact on financial aspects, through process improvement and waste reduction, not all of them effectively demonstrated the associated financial gains. Therefore, to provide greater visibility to the results obtained, they will be presented below in three charts: Chart 2 - Benefits/barriers to Lean Healthcare implementation^(7,11,15)^; Chart 3 - Economic aspects involving Lean Healthcare implementation^(12,14,16)^; and Chart 4 - Process improvements through Lean Healthcare implementation^(13,17-19)^.

**Chart 2:** Distribution of articles highlighting benefits/barriers to Lean Healthcare implementation according to citation, objective, design, outcomes and conclusions/recommendations, 2023

Benefits/barriers to Lean Healthcare implementation				
**Citation**	**Objective(s)**	**Design**	**Outcomes**	**Conclusions/recommendations**
^(11)^	Develop and refine a theory on Lean sustainability in pediatric healthcare.	Realistic review, with experiential approaches and literature searches. It established the hypothesis that the method in implementation and the resources provided shape the contexts and results for Lean sustainability. The term “pediatrics” and synonyms were searched in an EndNote database of 5,000 references, compiled from systematic review research on lean management in healthcare.	In summary, it was demonstrated that: - The degree of success or failure depends on the ways in which people “understand” Lean and align their values and organization’s values with this methodology’s values; - The engagement of different hierarchical levels and the degree of organizational involvement are essential; - There is a need for a systemic view of the complexity of Lean implementation and sustainability in healthcare.	There was a lack of studies that assessed the sustainability of the method, suggesting that future research examine whether implementation predictors are the same or different from sustainability and assess the underlying mechanisms that influence the sustainability of Lean in pediatric healthcare.
^(15)^	Analyze the scientific evidence in the literature on assessing Lean Healthcare after its implementation.	Integrative review, covering the period from 2008 to 2009. The following terms “Total Quality Management”, “Quality Improvement” and the keyword “Lean healthcare” were used in the search strategy. 126 articles were identified, 81 duplicates were excluded, leaving 49. Of these, 22 articles were eligible for full reading, and, after applying the inclusion and exclusion criteria, a sample of 18 articles was obtained.	The results were grouped into three evaluative categories: - Institutional contributions: promotes waste reduction, cost analysis, increased productivity, financial return; - Contributions from a professional perspective: provides participatory management with greater involvement of workers in decision-making processes; - Contributions to patients: increase satisfaction, favorable attitudes and behavior.	The need to establish, for management, a systematic method of monitoring the results achieved in the Lean Healthcare implementation phase was reinforced. Considering the relevance of value, in this method, when defined by patients, new research from this perspective could lead to new evidence.
^(7)^	Reflect on management practices that can be applied to hospital institutions with a view to achieving better care and financial results.	Reflective study approaching the themes: process-based management; the Lean Six Sigma methodology; continuous improvement; and value-based healthcare and cost management.	With the adoption of process-based management, Lean Six Sigma methodology, continuous improvement and value-based cost and health management, the following were evidenced: increased productivity and team efficiency; reduction in patient waiting time for care; standardization of care processes; cost reduction; improving teamwork; reduction in length of patient admission; increasing the quality of the service provided; increased patient satisfaction; increased patient and healthcare professional safety; and professional satisfaction.	The management practices studied have been well recognized. When used, processes can be improved, increasing efficiency, reducing waste, adding value to the business, increasing revenue and resulting in savings that can be passed on to consumers through improved quality.

**Chart 3:** Distribution of articles highlighting economic aspects involving Lean Healthcare implementation according to citation, objective, design, outcomes and conclusions/recommendations, 2023

Economic aspects involving Lean Healthcare implementation				
Citation	Objective(s)	Design	Outcomes	Conclusions/recommendations
^(12)^	Identify the economic potential of surgical instrument configuration to optimize the process in total knee arthroplasty (TKA).	Comparative study, carried out in the department of orthopedics, traumatology and plastic surgery of a university hospital in Germany, aiming to investigate the effects of changing a configuration of surgical instruments type A (standard set) to a configuration type B (set of specific size). Lean methodology was applied through standardization, increasing TKA process workflow and process mapping.	- Optimization in use of surgical trays (from six to five); - Reduction in the number of instruments (from 188 to 98); - Increase in the rate of use of surgical trays (from 26.33% to 50.64%); - Reduction IN sterilization cycles (from 116 hours and 30 minutes to 58 hours and 46 minutes); - Reduction in instrument preparation time by the Material and Sterilization Center (MSC) (50.00% when preparing orthopedic materials, 44.00% with packaging and checking); - Reduction in time for planning the operating room (13 minutes and 22 seconds/procedures, with 7 minutes and 30 seconds for preparing the instruments and 5 minutes and 52 seconds for cleaning the room); - There was a reduction in the total time of the TKA process (incision to suture) of 53 minutes and 28 seconds for every four procedures, allowing another procedure to be scheduled and resulting in an additional contribution margin (between €701 and €1,983 per day).	TKA time was saved by introducing a set of specific instruments. It allowed using the operating room for a greater number of procedures or other surgical procedures, generating additional revenue. It is suggested that cost-based assessments of benefits utilize full operational capacity so that any lost time is linked to opportunity costs.
^(14)^	Assess the costs of brachytherapy performed in an integrated imaging unit versus a distributed and decentralized workflow when performing precision intraoperative breast radiation therapy (PB-IORT).	Comparative study, carried out by the University of Virginia School of Medicine and Thomas Jefferson University. One institution performs the entire procedure in a brachytherapy suite, which contains an integrated imaging unit, allowing all steps to be performed in the same room without moving patients (model 1). In the other, breast-conserving surgery and radiotherapy occur in two different locations: an outpatient surgical room and a brachytherapy room (model 2). Process maps were created to describe each step and its deliverables.	- PB-IORT cost was lower in model 2 (institution with two facilities), US$3,262.22, when compared to PB-IORT cost in model 1 (institution with an integrated brachytherapy suite), US$3,996.01; - The difference resulted from the costs related to the human resources required (US$764.89 in model 2 versus US$1,263.41 in model 1), notably with the nursing and anesthetic team, as, in the institution, with the integrated set of brachytherapy, there was an increase in the time spent by the radiation oncology nursing team and more time in anesthetic care; - Equipment costs were also higher at the institution with the integrated brachytherapy set (US$332.60 versus US$97.33).	There was an increase in costs in model 1 (institution with an integrated brachytherapy suite), however cost differences must be weighed in relation to the potential impact on patients’ experience with these different models. Value-based care emphasizes outcomes over cost, and therefore further investigation is needed to weigh additional cost against patient satisfaction and overall outcomes.
^(16)^	Assess time-driven activity-based costing (TDABC) in interventional radiology for the treatment of guided vascular malformation.	Retrospective analysis performed at a tertiary hospital in Muenster, Germany. It encompassed two types of vascular malformation treatments, venous and arterial. All activities involving these two treatments were integrated through a process map, highlighting contributions from Lean methodology. Based on the findings, process improvement strategies would be created. TDABC was based on two parameters: operational and installed capacity; and time consumption of each resource.	- The study demonstrated that the time for arterial treatment (1,191 minutes) was longer than venous treatment (637 minutes); - The modification of the therapeutic process reduced staff time by 16.00% and 30.00%, and reduced costs by 5.50% in arterial treatment and 15.70% in venous treatment; - There was an increase in revenue through contract negotiations for specific materials for embolization or sclerotherapy; - Extra revenue combined with cost savings increased profits to 69.00% for venous treatment and 40.00% for arterial treatment.	TDABC facilitated the accurate calculation of the costs of interventional radiological treatment cycles and optimized internal processes, increasing cost reduction and revenue generation associated with the treatment of vascular malformation.

**Chart 4:** Distribution of articles showing process improvements through Lean Healthcare implementation according to citation, objective, design, outcomes and conclusions/recommendations, 2023

Process improvements through Lean Healthcare implementation				
Citation	Objective(s)	Design	Outcomes	Conclusions/recommendations
^(13)^	Assess the effect of a pre-sternotomy radial artery (RA) harvesting strategy on surgery time and costs.	Comparative study, carried out at the Stanford University School of Medicine. Thus, 41 patients underwent elective myocardial revascularization, for the first time, with extracorporeal circulation using two techniques (phases I and II) with different resources. Lean management concepts were used, with reference to improving workflows and operating room efficiency, resulting in shorter and less expensive surgeries.	- A reduction in the total surgery time (from 287 minutes and 5 seconds to 255 minutes and 5 seconds) and a reduction in the total time used in the operating room (from 380 minutes and 8 seconds to 145 minutes and 3 seconds) were identified; - After statistical analysis, an average saving in surgical time of 33 minutes and 3 seconds and an average total saving in surgery time of 35 minutes in phase II were revealed. - The strategy used in phase II requires only one endoscopic conduit collection kit to be used per operation, saving 8.0% of surgery-related costs per patient.	The strategy improves intraoperative workflow, reduces surgery time and cost, and advances the delivery of high-quality care to patients.
^(17)^	Understand which Lean healthcare tools are currently being used to review processes and find the results of implementing such tools highlighted in the literature.	Integrative literature review, covering from 2015 to 2019. The search strategy included the descriptors “Quality Management” and “Process Assessment (Health Care)”, as well as their synonyms in Portuguese and Spanish. All of them were associated with the keyword “Lean Healthcare” in the title or abstract. 240 articles were identified, and 123 duplicates were removed, and 100 were chosen which, after reading the full text, resulted in a sample of 33 articles.	The main results evidenced with Lean contributions were: - Time reduction: reduction of patient length of stay by 2.6%, reduction of length of admission from 20.6% to 17.8%, reduction of treatment time from 187 to 60 days, reduction of total operative time from 714 to 607 minutes, reduction in patient turnover time from 41 to 32 minutes, reduction in time between incision and application of surgical dressing from 81.5 to 71 minutes; - Cost reduction: increase of US$ 1.31 in medical prescriptions/minute and US$ 3.27 in hospital costs/minute, reduction in the number of incidents due to billing errors, non-insurance coverage and incorrect information by 99.6%, 75.0% reduction in overtime; - Workload reduction: team workload reduction by 70 minutes with a reversal of the time allocated for service by 9 minutes, reduction of distance traveled by automated infusion professionals by 14.6%, reduction of automated infusion tasks by 26 minutes per day; - Patient response time: increase in response time to patient care from 50.9% to 60.4%, increase in punctuality of care from 76.1% to 81.9%, increase in discharge prescriptions by 10 am from 15.6% to 47.1%, increase in effective patient discharge by 12 pm from 10.5% to 20.6%, reduction in time between request and catheter insertion from 3.74 to 1.89 days.	Using the main Lean tools has helped to improve processes in health services. However, although it is known that the Lean philosophy offers methods for applying and analyzing its results, it is important to highlight the importance of researchers properly designing their studies, with a sample size that supports scientific proof and the replicability of their applications.
^(18)^	Assess the application of process management using the PDCA tool (plan, do, check and act) in the daytime operation ward and its influence on nursing quality and safety.	Comparative study, between March 2019 and March 2020, with a routine management model, and between March 2020 and March 2021, with the application of PDCA, conducted at a science and technology university hospital in Huazhong, Wuhan, China, including a sample of 907 patients. Nursing quality was observed in both groups based on the dimensions: nursing safety, specialist quality and nursing standards; individual quality control (assessments twice a month); quality of nursing in a surgical center (SC) (assessment one day after the operation: equipment management; preparation of materials and cooperation between the team; incidence of adverse events and nursing errors; number of problems in medical record document management; patient satisfaction).	The PDCA cycle application led to improvements in the following areas: - Quality of nursing and nursing safety; - Improving the quality of nursing in the operating room, instrument management, instrument preparation, cooperation between nurses and disinfection; - Decreased occurrence of adverse events and nursing errors, incidence of nosocomial infection, iatrogenic injury, information verification error, equipment failure, violation of operating regulations, electrocardiogram monitoring error, infusion operation error and medication; - Improvement in number of problems on the temperature sheet, medical order, assessment sheet, nursing record, among other nursing documents; - Improvement in nursing communication, professional technology, nursing service attitude, nursing environment and education.	In summary, process management using PDCA, in the case group, compared to the control group, reduced the incidence of adverse events, non-conformities related to process management and nursing routines, providing improvements in nurses’ communication and educational attitudes (P<0.05).
^(19)^	Investigate how the disinfection center participates in sterilized item supervision and management in clinical departments.	Case study carried out at the Zhejiang University School of Medicine, Hangzhou-China, between July 2018 and June 2019. The routine covering the relationship between the MSC and the clinical departments was mapped, identifying the most frequent problems. Based on this scenario diagnosis, a standardized sterile clinical article management was incorporated, such as quality control, applied quarterly. MSC professionals inspected the storage environment for sterile items in 65 clinical departments. Assessments were carried out through on-site visits and control meetings with the inclusion of continuous improvement to support standardized management.	- The main problems encountered were in relation to sterile kits that were expired and not removed from the storage area and the number of packages inconsistent with records; - There was the standardization of aseptic items that contributed to reducing lost packaging and reducing costs (from 1,190 to 70 yuan) caused by the loss of sterile packaging.	By adopting a decentralized way of supervising and managing sterile materials, MSC provided targeted training and on-site guidance to correct existing problems. At the same time, it contributed to rational consumption associated with the loss of sterile packaging, reducing costs.

## DISCUSSION

The analysis of the sample studies of this integrative review showed the use of Lean methodology in applied research^(12-14,16,18-19)^ linked to university hospitals, places where the teaching, research and care tripod predominates. Interventions, that contribute to improving the efficiency, efficacy and effectiveness of the health services provided, which underlie the training of human resources and favor the conduct of health research using available, but not unlimited, resources, are needed.

Among the studies, 50.0% were published in American journals, however, when analyzing the countries that carried out the six (100.0%) applied research^(12-14,16,18-19)^, its geographic distribution was verified, with two (33.3%) conducted in China, two (33.3%) in Germany (33.3%) and two (33.3%) in the USA (33.3% ). In 90.0% of these studies, interventions were implemented in surgical units and 10% in units with a direct interface to them, in this case, MSC.

It is noteworthy that SC is considered one of the most complex and costly units, as it requires high technology to make procedures viable, in a hospital organization. Professionals who work in this unit need to develop skills to improve management processes, with the perspective of increasing and rationalizing the financial resources required.

A study^(20)^ carried out with nurses working in SC identified the essential competencies for nursing professionals in this area. One of the aspects highlighted was assertiveness in communication flow, including strategic meetings between the team to discuss, plan and assess work processes. With the aim of articulating administrative and clinical spheres, as well as strengthening the culture of cooperation and collaboration, this management model, admittedly, consists of a tool to qualify the care provided.

The findings of the aforementioned study^(20)^ are corroborated by the study^(11)^, which demonstrated the importance of engagement and alignment of people’s and the organization’s values with Lean methodology’s values, aiming to provide participatory management with greater involvement of workers in decision-making processes^(15)^, without losing sight of the strategic map and organizational goals.

From this perspective, Lean methodology adoption makes the use of strategic, tactical and operational indicators crucial, as it requires a clear definition of the objectives and goals to be achieved, with systematic and continuous measurements. Strategic map integration with this methodology enables a systemic view of the institution by identifying weaknesses and points that can be improved, seeking sustainability based on results indicators, with gains in the small, medium and long term^(7)^.

Results related to savings and earnings, predictors of Lean interventions, were only reported in three studies^(12,14,16)^. Two studies^(12,14)^, which had evidence of correlation with economic aspects, were carried out in SC, with variables linked to the general work process, but both present limitations regarding the techniques used by medical professionals, denoting a hypothetical barrier in action sustainability, since the results obtained were linked to people and not to processes. One of these studies^(14)^, despite demonstrating that Lean methodology application, as a change in the work process, generated financial burden, highlighted that it could be balanced when related to customer satisfaction.

Among the studies analyzed, there was no evidence of measuring the results of customer satisfaction, team satisfaction or engagement levels. Thus, a systematic review^(21)^, conducted in 2022, reflected on the need for studies with Lean methodology supported by digital technologies in health services, through computerized systems, such as automation, simulation, real-time location system, telemedicine, machine learning, among others. It indicated that, with the digital transformation experienced in recent years, there has been an exponential growth in data, and digital technologies are already being used to carry out descriptive, predictive and prescriptive analyzes. Machine learning has been used to predict resource needs, treatment outcomes, and readmission patterns. Medication dispensing can now be carried out through automated distribution, with surgical methods being carried out using robotics, ultraviolet disinfection systems carried out by robots to prevent infection, consisting of technological improvements that are gradually becoming part of the realities^(21)^. The analysis of the ten articles^(7,11-19)^ demonstrates that the interventions were related to mechanical actions in the work process, with no use of digital technologies being found.

It is worth emphasizing that the Lean philosophy does not dispense with scientifically based participatory management, which can bring intangible benefits to the quality of the work processes of healthcare teams. Quality science offers valuable paths for managing the hostility that inspections, awards and punishments can generate, and it is important that this topic integrates health team professional and manager training^(22)^.

Among the studies analyzed, three corresponded to review articles^(11,15,17)^, which assessed the Lean tools used in research^(17)^, the results after implementing Lean methodology^(16)^ and its sustainability^(11)^. In this integrative review, it was found that the Lean methodology can bring benefits to organizations^(7,15)^, and is associated with process improvements, waste reduction, increased efficiency and productivity, improvement in service quality^(13,17-19)^, contributing favorably to economic aspects^(12,16)^.

Three articles^(11,15,17)^ presented outcomes that correlated the Lean methodology’s success or failure to behavioral attitudes, whether of the team or leadership. Given this context, the lack of clarity regarding its implementation to overcome the barrier of local units and materialize, in a sustainable manner, as a management philosophy at all hierarchical levels becomes evident. In this regard, one of the challenges of adopting the Lean method is employees’ resistance in relation to change strategies, demanding the breaking of institutional paradigms, through process changes, changing culture, working with systemic objectives, team engagement and continuous improvement^(10)^. Thus, this method can be implemented in a sustainable way aiming at the continuous improvement of work processes in health and nursing, with favorable repercussions on associated costs.

### Study limitations

As a limitation of this study, only the use of free access articles is indicated, which may have had an impact on sample size.

### Contributions to nursing, health or public policies

This study demonstrated that Lean methodology has been widely disseminated to identify value for end customers and eliminate losses. Its application, adapted to health services, has been the object of study in different realities. It involves mapping the value chain and disseminating philosophy at all hierarchical levels, bringing together senior management professionals (strategic level) and front-line professionals (operational level) working with patients.

## CONCLUSIONS

In this integrative review, based on the analysis of ten articles, it was found that only three of them effectively investigated the financial aspects associated with Lean methodology application. The others only mentioned the possibility of financial gains through process improvements and waste reduction.

For future studies, research related to direct labor cost reduction in different contexts of health service provision is suggested, through improvement implementation in work processes using Lean methodology.
